# Experimental and Theoretical Study of Multifilamentary Resistive Switching in Nanoscale Transition Metal Oxide Films

**DOI:** 10.1021/acsomega.5c06052

**Published:** 2025-09-08

**Authors:** Mikhail I. Fedotov, Ekaterina V. Klyukina, Aleksandr S. Vankaev, Andrei G. Znamenskii, Sergei V. Koveshnikov

**Affiliations:** † 54744 Institute of Microelectronics Technology and High-Purity Materials of Russian Academy of Sciences, 6, Academician Osip’yan Str., 142432 Chernogolovka, Moscow Region, Russian Federation; ‡ Sechenov First Moscow State Medical University, Trubetskaya Street, 8, building 2, 119991 Moscow, Russian Federation; § Russian Technological University MIREA, 78 Vernadsky Avenue, 119454 Moscow, Russian Federation

## Abstract

The development of metal oxide based resistive random-access memory is of the greatest importance for creating a new class of memory in various applications, including large-scale memory arrays, analogue neuromorphic computing systems, and energy-efficient system-on-chips. The filamentary switching mechanism of resistive memory provides great scalability, while its nonvolatility, energy efficiency, high retention, and write/erase speeds make resistive memory a prospective universal memory. The ability of resistive memory to mimic the behavior of biological synapses coupled with its multilevel storage capabilities makes this novel type of memory a suitable hardware for analogue neuromorphic systems. However, practical application for large-volume memory arrays for neuromorphic systems is limited by unresolved fundamental issues including intrinsic cycle-to-cycle variability of a high resistance state and extrinsic cell-to-cell variability of switching from a low to a high resistive state. Mitigation of these issues is impossible without a clear understanding of physical processes occurring inside dielectric layers of resistive memory. In this paper, we address these issues via theoretical and experimental investigation and demonstrate that both issues are related to the formation of multiple conductive filaments.

## Graphical abstract

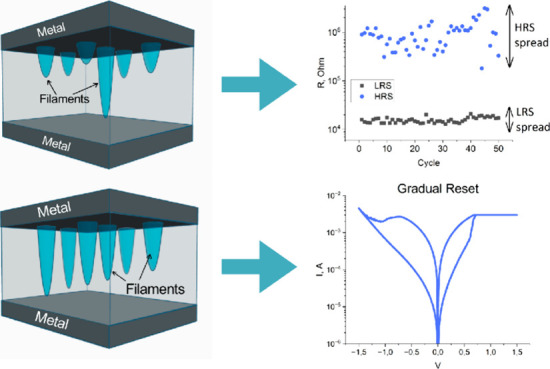

## I Introduction

Neuromorphic computing, in which electronic devices mimic the work of biological synapses, will play an important role in the near future for the creation of new types of computers.^1^ Traditional methods of simulating the work of biological synapses and building neural networks require a large number of transistors, memory elements, and significant time for information processing.^2^ The limitations of the traditional digital neural networks can be overcome with the help of analogue neuromorphic systems in which synaptic switching is implemented, not algorithmically but physically, based on the processes in certain novel devices.^1–4^ One of the most promising options for the component base for analogue neuromorphic systems is resistive random-access memory (RRAM).^4–11^ The main advantages of RRAM include reliability, scalability, a large number of switching cycles, and, most importantly, the possibility to achieve multilevel resistive switching, owing to which the RRAM cell can work as an artificial synapse.^9–11^ RRAM arrays with improved functionality and reliability are of great importance for the development of new, more efficient elements needed to develop and fabricate neuromorphic devices.

Despite significant progress in the development of the two- and multilevel elements of metal oxide based resistive memory, their practical application for large-volume memory arrays for neuromorphic systems is limited by a number of unresolved fundamental problems.^12^ First, it is necessary to achieve a clear understanding of the physical processes occurring in the dielectric during its transition from a high resistance state to a low resistance state and vice versa. Resistive switching in the transition metal oxides is generally believed to be due to the formation and disruption of nanoscale conductive filaments (Figure 1).^10^ During switching from a high-resistance to a low-resistance state (Set), a conductive filament (CF) within a dielectric is formed due to drift of oxygen vacancies forced by an external voltage applied to metal electrodes.^10^ In the reverse process of switching from a low-resistance state to a high-resistance state (Reset), the filament is oxidized due to oxygen diffusion, which is enhanced by Joule heating of the filament. The main evidence of the filamentary switching mechanism is a very weak, if any, dependence of the conductivity of the RRAM cell on its area.^10^

**Figure 1:**
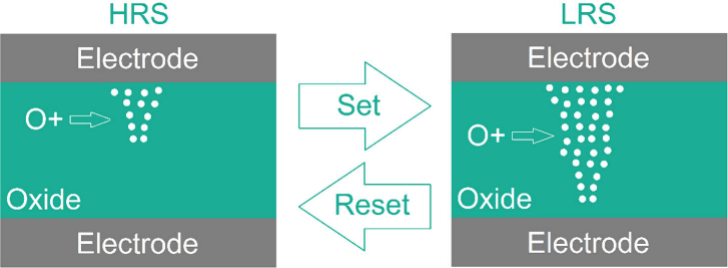
Schematic diagram of resistive switching (Set and Reset processes) in transition metal oxide RRAM.

Multilevel switching is achieved via controlling the voltage magnitude during the transition back from a low-resistance to a high-resistance state (Reset).^12^ As a result, one low-resistance and several high-resistance logical states can be achieved in the cell due to a change in the length of the conductive filament. One of the key problems limiting the number of resistive states of a RRAM cell is a low reproducibility of the resistance in a high-resistance state.^12,13^ Another crucial problem is that the transition from a low-resistance state to a high-resistance state can be either sharp (sharp Reset) or smooth (gradual Reset) for the memory cells placed on the same chip. The physical mechanism of a gradual Reset remains the subject of discussion and intensive research.^14–19^ To create RRAM-based analogue-like neuromorphic systems, it is necessary to achieve reliable multilevel switching, for which a gradual Reset is a key factor. Therefore, the elucidation of the physical mechanism responsible for the smooth change of the current flowing through the resistive memory element in a Reset mode is an important scientific task. Understanding the physical mechanism responsible for gradual Reset will allow creation of RRAM arrays with specified and controlled physical properties.

In this work, we conducted studies of RRAM cells based on two transition metal oxides—hafnium oxide and titanium oxide. On hafnium-oxide-based RRAM cells, the nonreproducibility of resistance in low- and high-resistance states was studied theoretically and experimentally depending on the maximum voltage in Reset mode. The process of smooth Reset was studied on titanium-oxide-based RRAM cells. We have obtained the data demonstrating achievement of several reliable resistive states owing to enforced gradual Reset. To explain the phenomena of low reproducibility of the resistance in a high-resistance state from cycle-to-cycle and of smooth transition from a low to a high resistance state during Reset, we have developed a model of multifilament resistive switching. We assume that not one but several conductive filaments of different lengths can be formed in a single RRAM cell. The very possibility of the occurrence of several conductive filaments in one memory cell follows both from our previous work^12^ and from the experimental results of other research groups.^13,20–22^ Depending on the relative difference of the lengths of the filaments, our model of multifilament switching allows us to explain both the nonreproducibility of resistance in the high-resistance state and the effect of smooth switching in a Reset mode. The effect of several filaments on the reproducibility of resistance in a high-resistance state and on a smooth Reset has also been experimentally verified.

## II Intrinsic Cycle-to-Cycle RRAM Resistance Variability in a High Resistance State

### ΙΙa Materials and Methods

Measurements of the parameters of resistive switching at direct current (DC) and statistical analysis of their intrinsic variability in hafnium-oxide-based RRAM were carried out using the 1T1R structure (1 transistor, 1 resistor) shown in Figure 2. The RRAM cell area is 4 μm^2^. The RRAM cell was connected in series with a MOSFET, which controlled the maximum (compliant) current in the cell during SET operation. The RRAM cell itself consists of a 10 nm layer of HfO_2_ deposited onto a TiN electrode by atomic layer deposition (ALD) and an 8 nm Ti layer placed by magnetron sputtering. The choice of this structure was driven by the need to create a nonstoichiometric HfO_*x*_ near the upper TiN electrode with an excess of oxygen vacancies. TiN electrodes were formed by magnetron sputtering.

**Figure 2:**
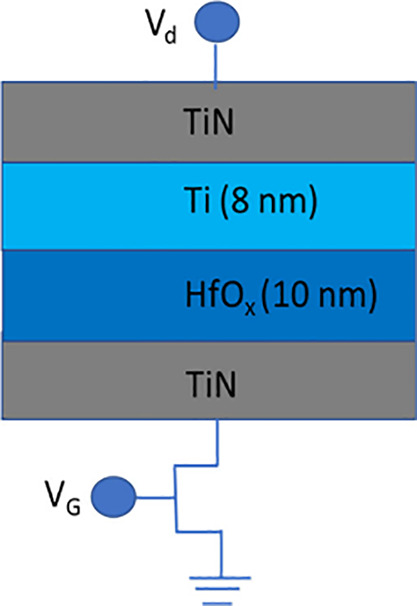
Schematic diagram of the 1T1R RRAM cell and the memristor cross-section structure.

During the forming process of the conductive filament, the maximum current flowing through the cell was limited to 18 μA. The maximum value of the negative voltage during the RESET process was 1.7 V. The variation in the characteristics of the studied cells due to external factors was negligible, with a standard deviation to mean resistance ratio less than 5%. Randomly selected 1T1R cells were subjected to a series of 50 switching cycles. The maximum voltage in a Reset mode had 5 different values (−1.7 V, −1.5 V, −1.3 V, −1.1 V, and −0.9 V), while the maximum current limit in a Set mode was 18 μA. The parameters of the same device were measured during 50 switching cycles for each of the 5 Reset voltages.

### ΙΙb Results

The current–voltage characteristics at various maximum voltages in the Reset mode are shown in Figure 3 (a, b, c, and d). The transition from a high-resistance to a low-resistance state occurs at a positive voltage and back at a negative voltage. Thus, resistive switching is bipolar in nature. The measured resistance spread in low- and high-resistance states for 50 consecutive cycles during Reset at various maximum voltages is shown in Figure 4(a) and Figure 4(b).

**Figure 3:**
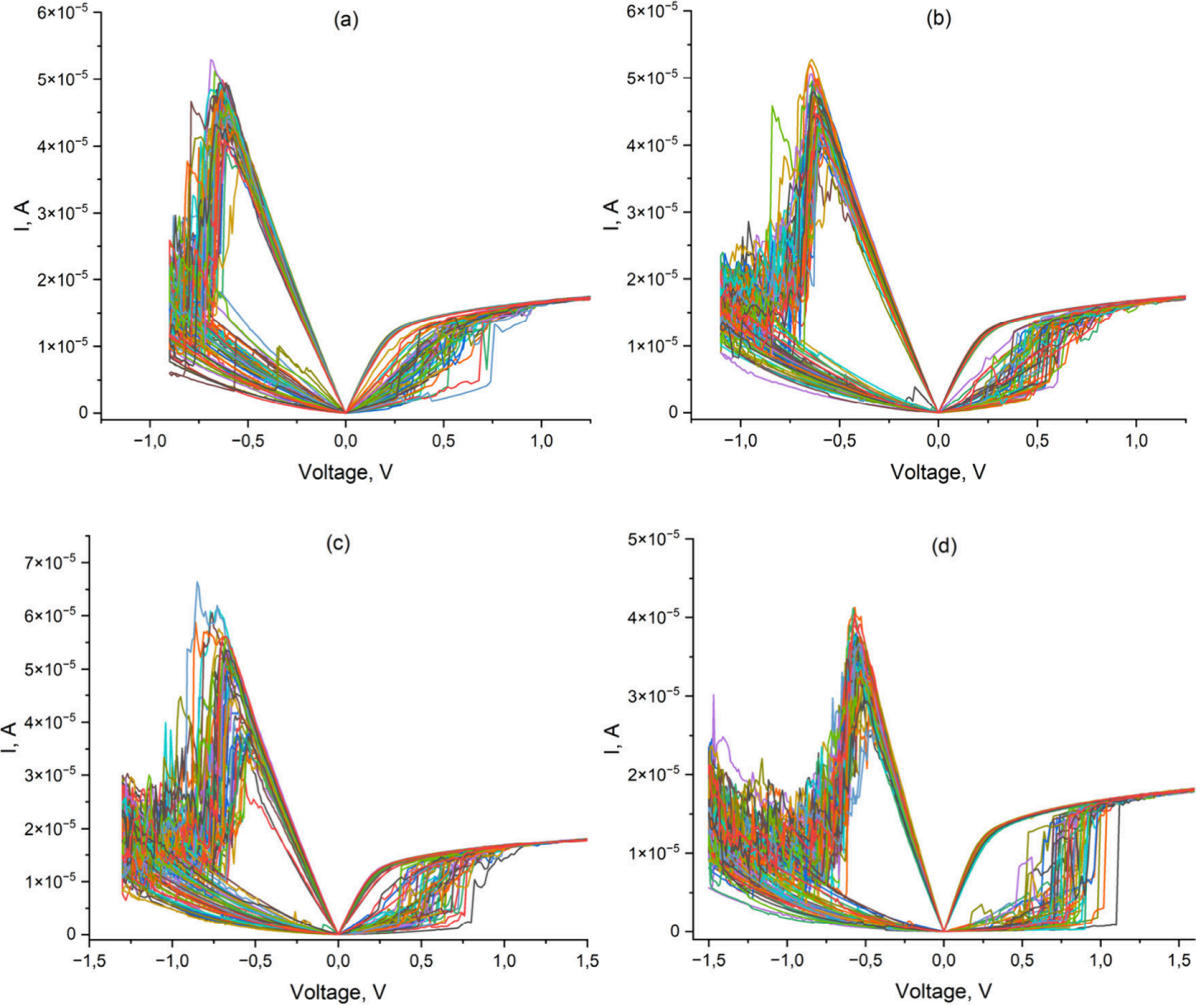
Current–voltage characteristics of HfO_*x*_ RRAM with current limitation in Set of 18 μA and maximum Reset voltages of 0.9 V (a), 1.1 V (b), 1.3 V (c), and 1.5 V (d).

**Figure 4:**
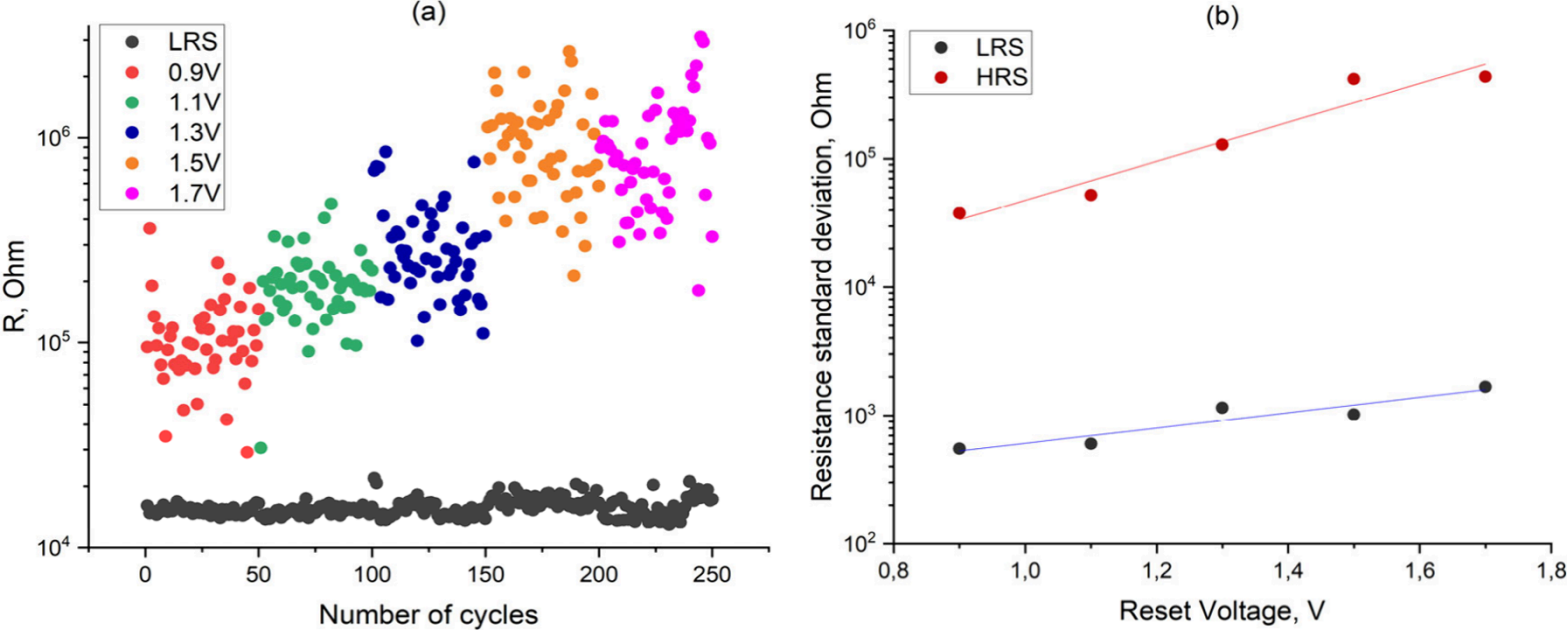
(a) Measured cell resistance spread in low-resistance (LRS) and high-resistance (HRS) states at different RESET voltages and at a maximum current of 18 μA. (b) Standard deviation of the resistance in the high-resistance state at different voltages in Reset.

Based on the data obtained during the experiment, we observe several clear dependencies. First, the resistance in the high-resistance state increases with an increase of the maximum voltage in Reset (Figure 4a). Second, the spread of resistance in the high-resistance state increases with increasing voltage in Reset, whereas the standard deviation for both low and high resistances in the low- and high-resistance states exponentially increases with an increase of the maximum Reset voltage (Figure 4b). Third, the resistance in the low-resistance state at some values of the voltage in Reset demonstrates a bimodal distribution (Figure 4a). As can be seen from Figure 4a, during switching at a Reset voltage of −1.7 V, the resistance value in the low-resistance state suddenly increased after about 35 cycles. The resistance spread also increased. When the Reset voltage was changed to −1.5 V, the resistance in the low-resistance state remained at a higher level and then decreased again at −1.3 V. The observed bimodal distribution of resistance in the low-resistance state may indicate the presence of additional conductive filaments in the low-resistance state.

To provide a physical explanation for the nonreproducibility of the resistance in low- and high-resistance states observed during the experiment, we propose a hypothesis of a cumulative action of several conductive filaments formed during resistive switching. Due to the physical roughness of the dielectric/electrode interface^21^ and possible nonuniform distribution of oxygen vacancies in the oxide, multiple conductive filaments can be formed simultaneously under the action of an external electric field during forming and/or during consecutive switching cycles. Although the conductivity in a resistive memory cell is controlled predominantly by a dominant (primary) filament, the smaller secondary filaments can slowly grow during subsequent switching cycles and contribute to the observed nonreproducibility of key switching parameters. The observed bimodal distribution of the resistance in a low-resistance state can also be caused by the growth of additional filaments, although their contribution to the resistance spread in the low-resistance state seems to be insignificant compared to their impact on the resistance spread in the high-resistance state (Figure 4). This may be due to the relatively short length of these additional filaments as compared to the length of the main filament in a low-resistance state. On the contrary, in the high-resistance state their lengths become comparable with the length of the dominant filament (Figure 5). Thus, the number and lengths of conductive filaments varying from cycle to cycle lead to a significant spread of the resistance.

**Figure 5:**
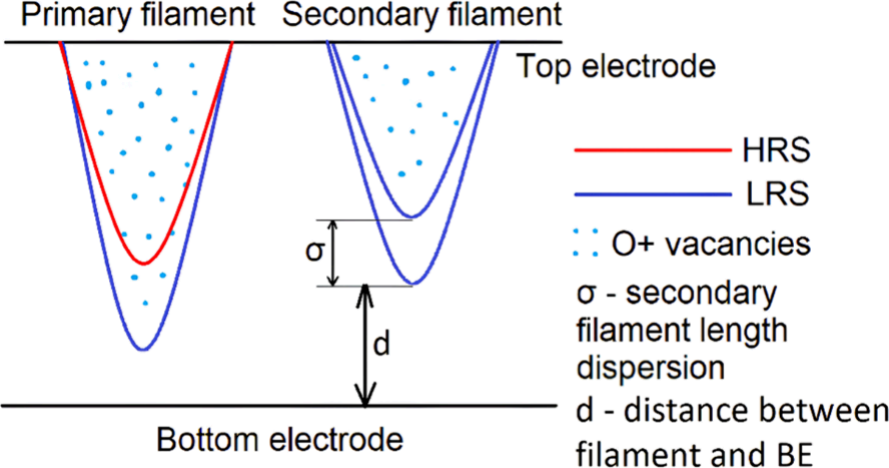
Schematic figure of the multifilamentary switching model.

The lifetime of both primary and secondary filaments depends on the voltage sweep rate.^23^ Higher voltage sweep rate results in faster switching in both Set and Reset modes; thus, the filament lifetime is equal to the switching time during transition between LRS and HRS (Reset mode). In the experiment discussed in this section, all measurements were conducted in DC mode with filament lifetime being equal to dozens of milliseconds.

To simplify the simulation, multiple additional filaments are replaced by a single side filament, which gives an equivalent spread of switching characteristics due to a random oscillation of its length within a certain limit (Figure 5). Due to the discrete nature of the defects (oxygen vacancies) which are involved in the formation of the main filament, its effective length also varies,^13^ but in a much smaller limit—by an order of magnitude less than the side filament (Table 1).

**Table 1:** Simulation Parameters

*V*_Reset_	Main filament length, nm	Main filament spread, nm	Secondary filament length, nm	Secondary filament spread, nm	Main filament cross-section, μm^2^	Secondary filament cross-section, μm^2^
0.9	9.15	0.05	8.5	0.5	0.01	0.01
1.1	9.1					
1.3	9.05					
1.5	9.0					
1.7	8.95					

The model assumes that the conductivity in the HfO_*x*_ thin films occurs due to direct tunneling^24^ between the conductive filament and the lower electrode, and the conductive filament itself has a negligible resistance compared to the resistance of the dielectric layer between the filament and the lower electrode. As neither the primary nor the secondary conducting filaments bridge the electrodes, the LRS resistance is determined solely by the gap between the longest (primary) filament and the lower electrode. During the Reset process, the Joule heating causes oxygen diffusion from the bulk oxide into the primary filament. This filament oxidation process increases the gap between the primary filament and the lower electrode and continues until the gap between the primary filament and the lower electrodes becomes comparable to the gap between the secondary filaments and the lower electrode. Due to the uncontrollable nature of the secondary filaments, they produce a significant contribution to HRS conductivity, which causes increased HRS resistance spread as compared to the LRS resistance spread. Although the film roughness is an order of magnitude higher than variation in the filament length, the key factor that determines the total resistivity of a RRAM cell is the gap between the filament and the lower electrode due to the tunneling nature of RRAM conductivity.

The assumption about the nature of the conductivity is based on the shape of the calculated potential barrier, which has a rectangular shape for switching voltages of up to 0.8 V (real conditions in the experiment). As can be seen from Figure 3, the switching voltage from the low-resistance to the high-resistance state ranges from 0.25 to 1.1 V depending on the voltage in the Reset. Thus, the height of the potential TiN/HfO_*x*_ barrier is 1.8 eV,^25^ which is much higher than the switching voltage. The thickness of the potential barrier is determined by the distance between the end of the conductive filament and the lower electrode. When a reverse voltage is applied during Reset, the conductive filament is oxidized, which leads to an increase in the thickness of the potential barrier. At lower Reset voltages, the barrier continues to remain rectangular, indicating forward tunneling as the dominant switching mechanism. If we consider the thicknesses of the conductive filaments to be set at a reading voltage of 0.1 V, the tunnel currents flowing through the RRAM cell at voltages not exceeding the height of the potential barrier 0 < *V* ≤ φ are determined by eqs 1–3:^24^

1 $$J=J_{0}\left({\left(\varphi -\frac{eV}{2}\right)}^{*}\exp\left(-A\sqrt{\varphi -\frac{eV}{2}}\right)-{\left(\varphi +\frac{eV}{2}\right)}^{*}\exp\left(-A\sqrt{\varphi +\frac{eV}{2}}\right)\right)$$

2 $$J_{0}=\frac{e}{2\pi hd^{2}}$$

3 $$A=\frac{4\pi d\sqrt{2m}}{h}$$

where *J* is current density, φ is potential barrier height, *e* is electron charge, *V* is applied voltage, *h* is Planck’s constant, *d* is dielectric thickness, and *m* is electron’s effective mass. The result of modeling the resistance spread in low- and high-resistance states is shown in Figure 6. Modeling was performed using the following variable parameters: the lengths of the main and secondary filaments, their diameter, and the spread of the length of the additional filament. The simulation parameters are listed in Table 1. The simulation results are in good agreement with the experimental results (Figure 6).

**Figure 6:**
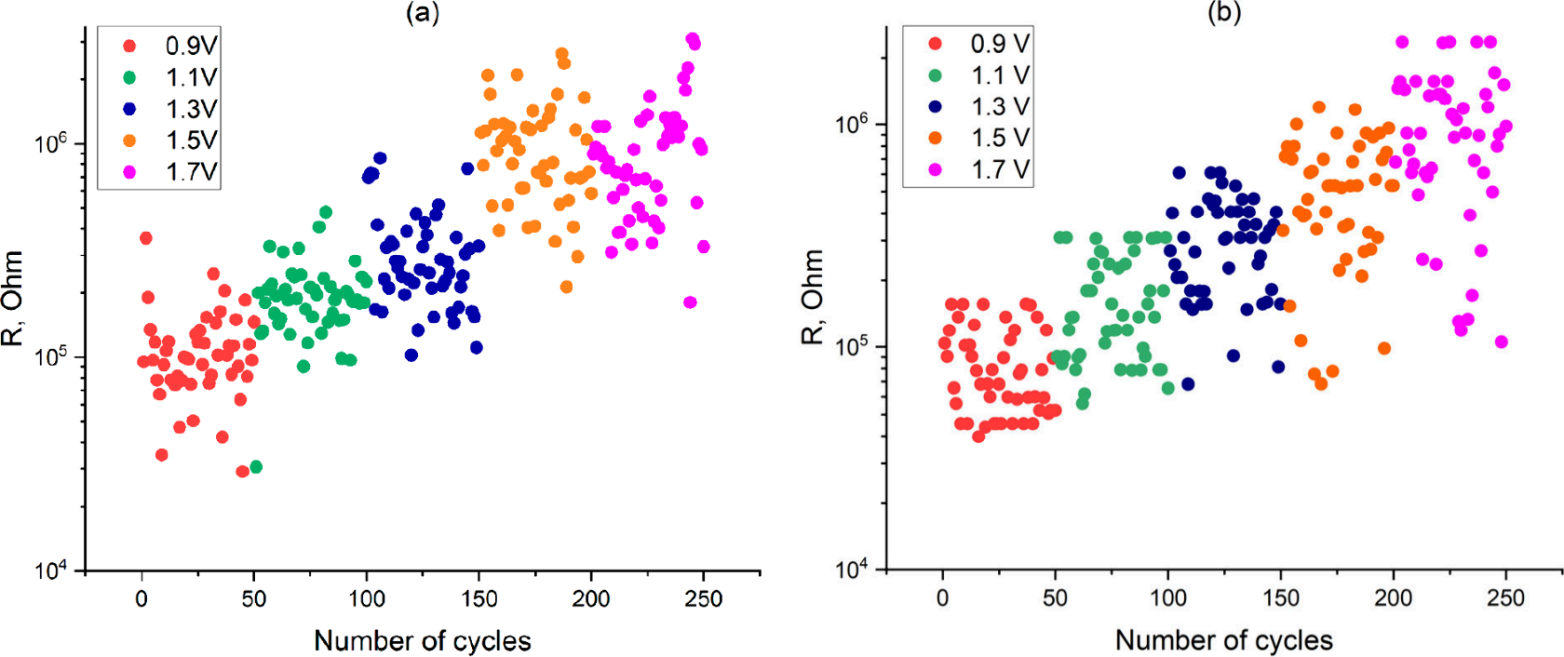
Experimentally measured (a) and modeled (b) resistance spread in the high-resistance state at different maximum Reset voltages.

The simulation results show that the filament length linearly decreases with increasing voltage in Reset, which leads to a larger spread of the resistance and to an exponential dependence of the standard deviation of resistance on the voltage in Reset (Figure 7). The presence of an additional filament with a large spread of length provides an additional contribution to the resistance variability at high voltages in Reset (Figure 6) since the lengths of the main and secondary filaments become comparable to such an extent that the division of filaments into main and secondary loses its meaning, and their electrical activity and contribution to the cell resistance become comparable. The dependence of the standard deviation of resistance on voltage in Reset is identical within the error limits (Table 2, Figure 7), which confirms the feasibility of the multifilamentary switching model described above. As the multifilamentary switching model requires a minimal number of initial assumptions (the main assumption is the spread of primary and secondary filaments, which induces the HRS resistance volatility), this model provides a good analytical description of the resistive switching process in thin HfO_*x*_ films. However, note that this model is inapplicable to the study of RRAM conductivity in the low-resistance state: it does not explain the bimodal resistance distribution in LRS observed in the experiment.

**Figure 7:**
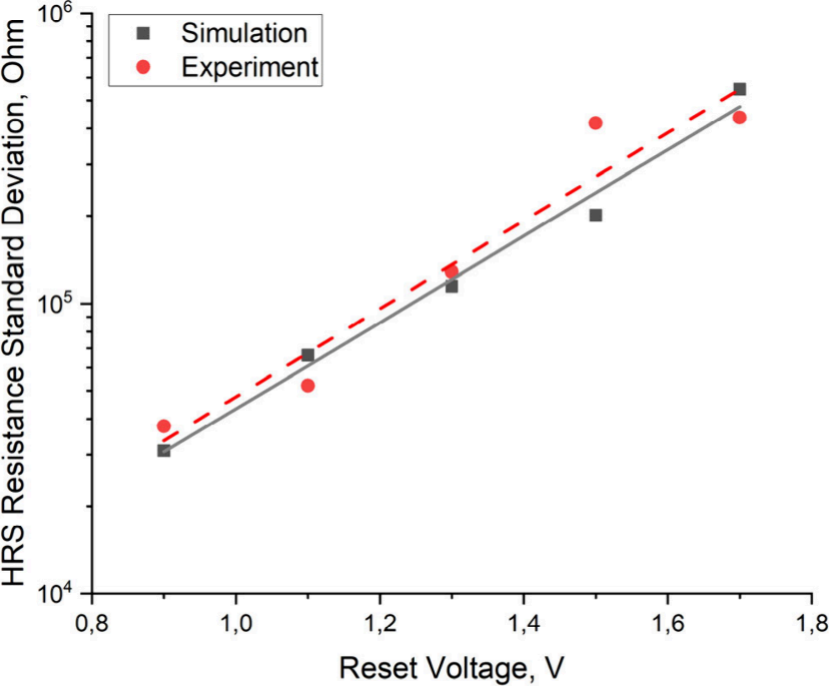
Comparison of the experimentally measured and simulated dependence of the standard deviation of resistance on the maximum voltage in Reset in a high-resistance state.

**Table 2:** Parameters Used for Approximation of Experimental and Measured Data Are Given in Figure 6

Equation	*y* = exp(*ax* + *b*)	
Plot	Experiment	Simulation^1^
*b*	3.16242 ± 0.2971	3.14822 ± 0.13018
*a*	1.51548 ± 0.22331	1.48805 ± 0.09785
Residual Sum of Squares	0.05984	0.01149
Pearson’s *r*	0.96894	0.99358
*R*-Square (COD)	0.93884	0.98719
Adjusted *R*-Square	0.91846	0.98293

## III Gradual Reset in Transition Metal Oxide RRAM

### ΙΙIa Materials and Methods

A bilayer structure of nonstoichiometric and stoichiometric titanium oxide was formed by magnetron sputtering on an oxidized silicon substrate covered with a platinum layer serving as a bottom electrode, while the top electrode was formed by tungsten. The TiO_*x*_ and TiO_2_ layer thicknesses were 15 and 35 nm; the top electrode area was 100 μm^2^. The switching current–voltage characteristics were measured by using a Keithley 4200A-SCS parametric analyzer. The Set cycles were performed by changing the DC voltage from 0 V to a positive value of 2 V in increments of 0.001 V. The maximum current was limited in the range of 500 μA–10 mA.

### ΙIΙb Results

The titanium-oxide-based RRAM cells demonstrated stable bipolar resistive switching with a resistance change of more than 2 orders of magnitude (Figure 8). The structure was subjected to a series of measurements in which several values of the maximum negative voltage were used during Reset to achieve a smooth transition from a low to a high resistance state, as shown in Figure 8. As can be seen, at least 6 intermediate resistive states were obtained.

**Figure 8:**
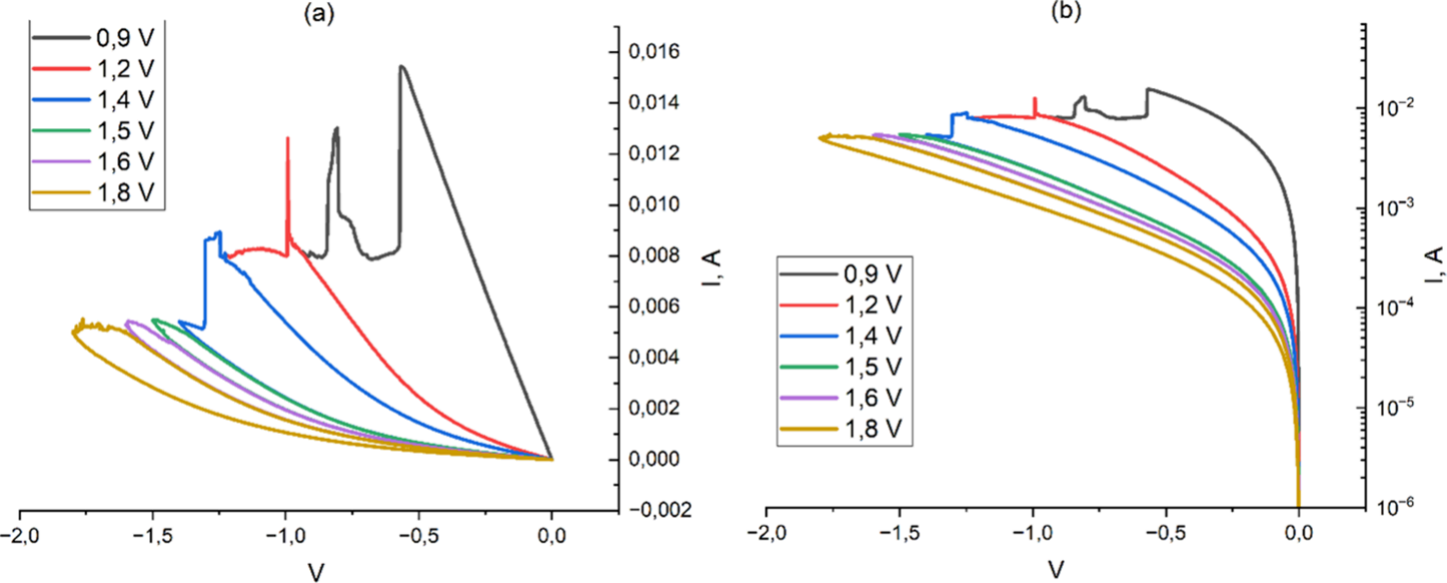
Current–voltage characteristics of titanium-oxide-based RRAM at multistage voltage change in a Reset mode on a linear (a) and logarithmic (b) scale.

To explain the experimental results, a mathematical model of gradual switching in Reset mode was created. The phenomenon of gradual Reset is presumably associated with the presence of several parallel conductive filaments. To simplify calculations, each filament is represented as a conductive electrode with a dielectric gap between its end and the lower electrode (Figures 9–14). In the dielectric layer, a tunneling current flows through a rectangular potential barrier, the density of which is calculated according to the well-known direct tunneling formula.^24^ Since the height of the potential barrier between TiO_*x*_ and the electrodes in the experimental structure exceeds 1 eV,^25^ smoothing of the walls of the potential barrier due to image forces was not taken into account. For each filament, represented as a cylinder with a radius of 100 nm (this radius was selected based on the analysis of other studies on titanium-oxide-based RRAM^26^), the current strength was calculated depending on the thickness of the dielectric barrier and the applied voltage. Within the framework of the model, it was assumed that there are 5 parallel filaments with different lengths (Figure 9–14), created during Forming and/or Set processes. As the temperature of the filament increases when a negative voltage is applied and the current is growing prior to transition to a high resistance state, some of the heat is transferred to the surrounding titanium oxide layer, thereby leading to accelerated diffusion of oxygen, which is embedded in the vacant sites within the filament. As a result, the longest filament is oxidized, and its length is reduced. As soon as the lengths of two or more filaments become equal, further oxidation of these filaments occurs at the same rate of 0.05 nm/V (Figure 9), 0.075 nm/V (Figure 11), and 0.1 nm/V (Figure 13). Figures 10, 12, and 14 indicate the initial lengths of conducting filaments for Figure 9, Figure 11, and Figure 13, respectively. Since the current through each filament strongly depends on its length (and, as a result, on the thickness of the dielectric gap), with increasing voltage in Reset, more and more filaments participate in current conduction, which provides a smooth change of the RRAM cell conductivity. The temperature at which the oxygen diffusion coefficient in TiO_*x*_ depends was calculated based on the solution of the thermal conductivity equation for a filament with a cylindrical geometry.^27^ The thermal conductivity coefficient for thin titanium oxide film was assumed to be 0.7 W m^–1^ K^–1^.^28^ Thus, a theoretical model of a smooth Reset provided good agreement with the experimental data.

**Figure 9:**
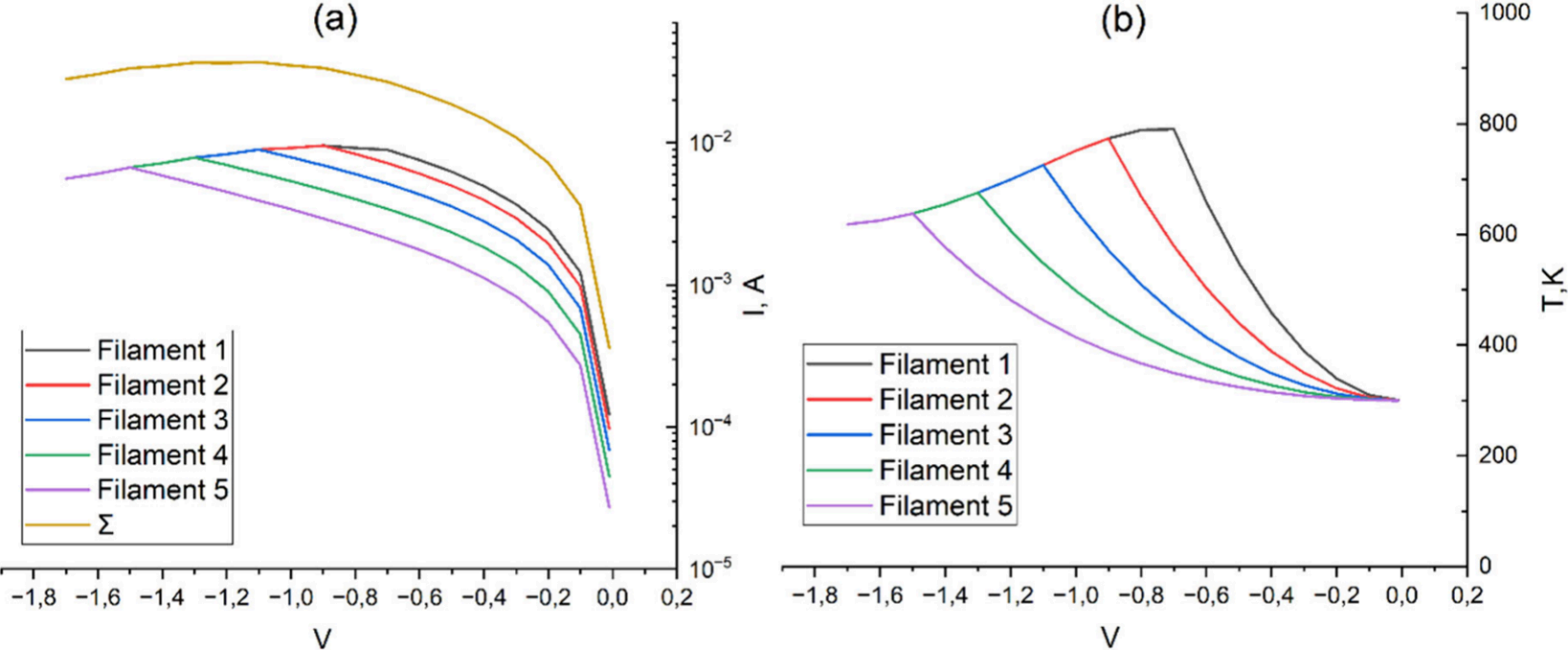
Current–voltage (a) and temperature–voltage (b) characteristics of the titanium oxide cell at a rate of filament oxidation of 0.05 nm/V.

**Figure 10:**
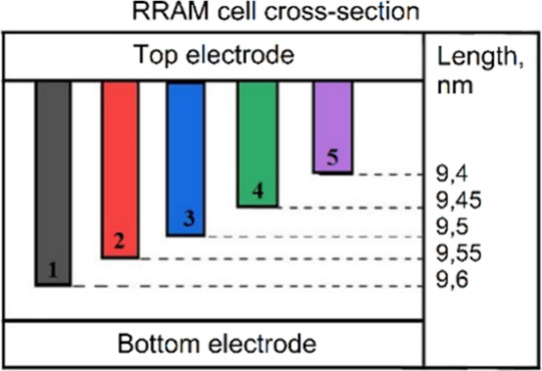
RRAM cross-section with initial filament length for simulation in Figure 9.

**Figure 11:**
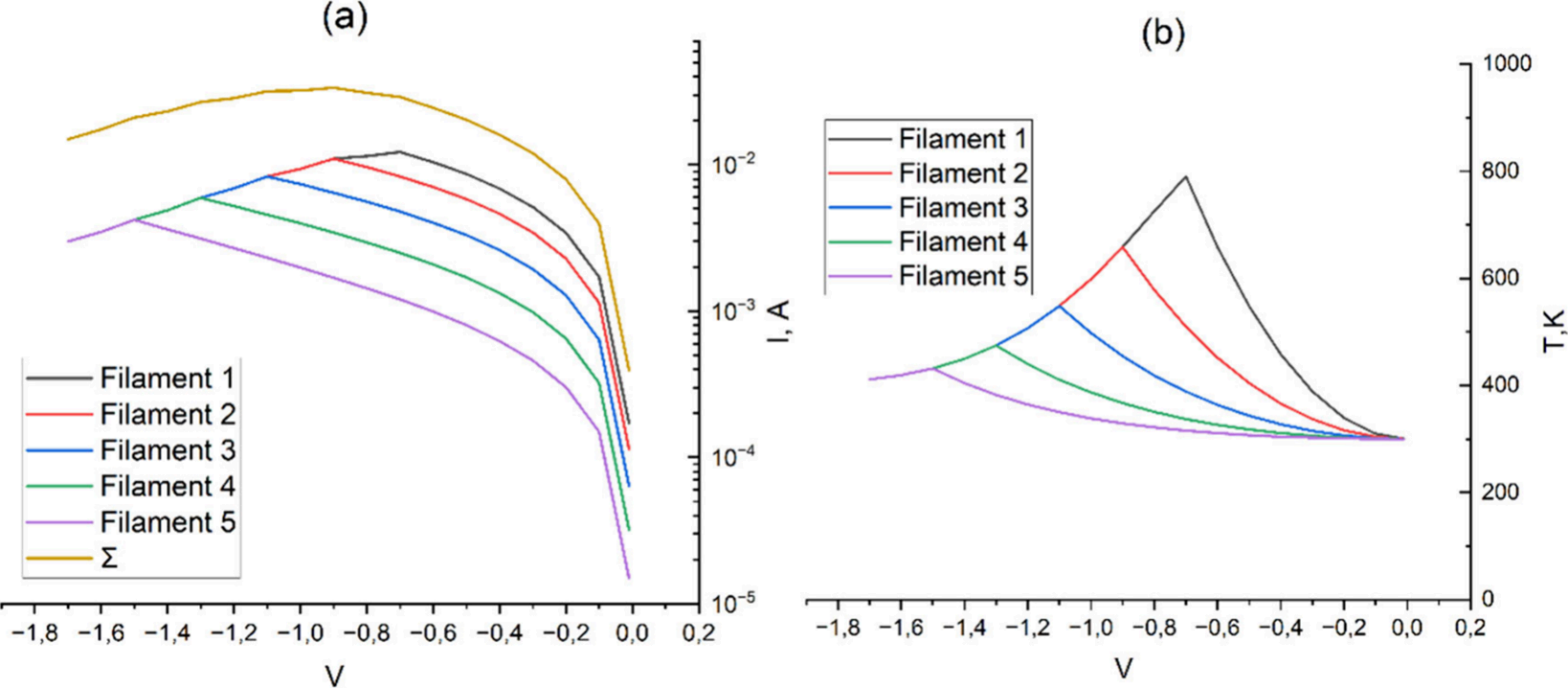
Current–voltage (a) and temperature–voltage (b) characteristics of the titanium oxide cell at a rate of filament oxidation of 0.075 nm/V.

**Figure 12:**
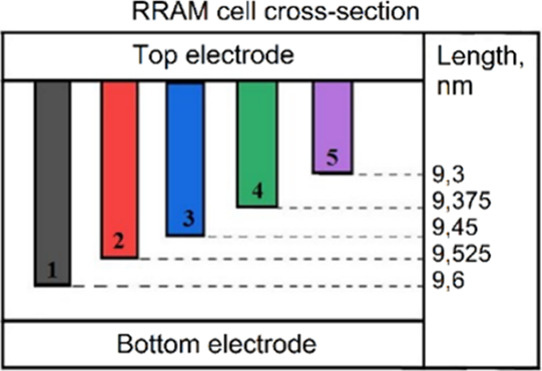
RRAM cross-section with initial filament length for simulation in Figure 11.

**Figure 13:**
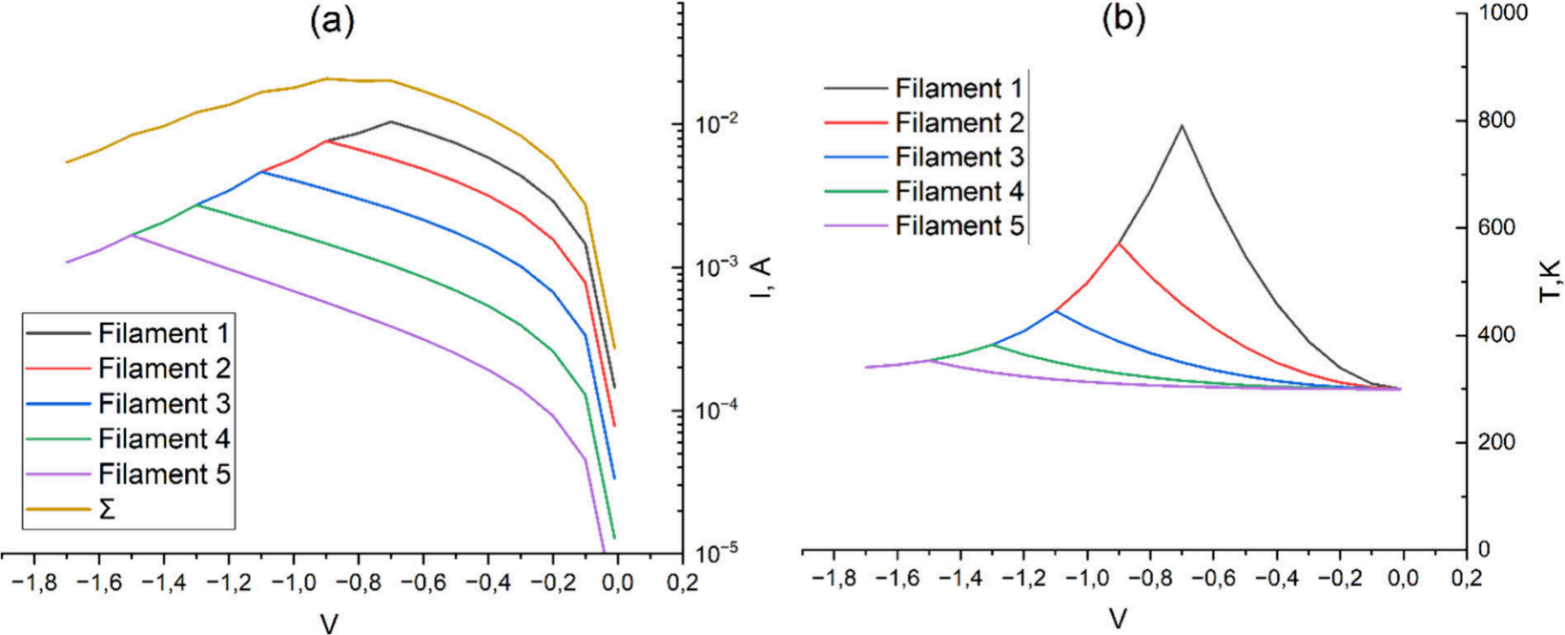
Current–voltage (a) and temperature–voltage (b) characteristics of the titanium oxide cell at a rate of filament oxidation of 0.1 nm/V.

**Figure 14:**
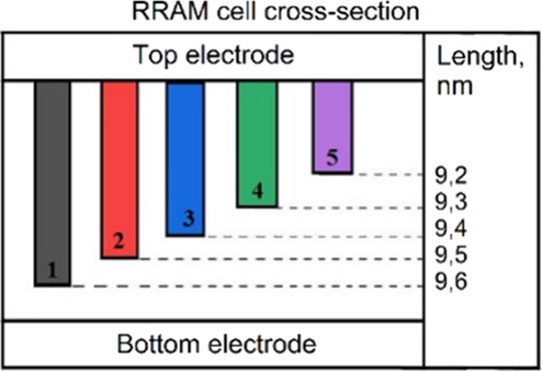
RRAM cross-section with initial filament length for simulation in Figure 13.

The presence of sudden current surges (Figure 8a) in the Reset mode can also be considered as evidence of the presence of several conductive filaments in the titanium oxide RRAM cell. Indeed, as the voltage in Reset increases, due to possible nonstoichiometry near the bottom electrode, an increase in a counter-directional filament near the bottom electrode is quite possible due to the change of the direction of the electric field. A high value of the electric field between the counter-directed filaments will lead to an increase in the current between them. This, in turn, will lead to an increase of the temperature and acceleration of oxygen diffusion, which will limit the growth of the lower filament and allow the Reset process to go further. Since the magnitude of the tunnel current strongly depends on the thickness of the potential barrier between the filament and the bottom electrode, the growth and decline of the current in such a situation is abrupt, as can be seen from Figure 8.

To experimentally verify the feasibility of the proposed model, an experiment on several HfO_*x*_-based RRAM cells was carried out. For this purpose, 3 RRAM cells with abrupt switching in a Reset mode were selected (Figure 15), indicating that each cell had one dominating conductive filament. During the Set step, the maximum current for these cells was different to form filaments of different length (Figure 15(a)). After connecting them in parallel, we observed smooth switching and were able to achieve several intermediate states (Figure 15(b)). The experimental results are found to be in good agreement with the theoretical results. In this way, the proposed model of gradual Reset driven by multiple filaments within a RRAM cell was experimentally verified.

**Figure 15:**
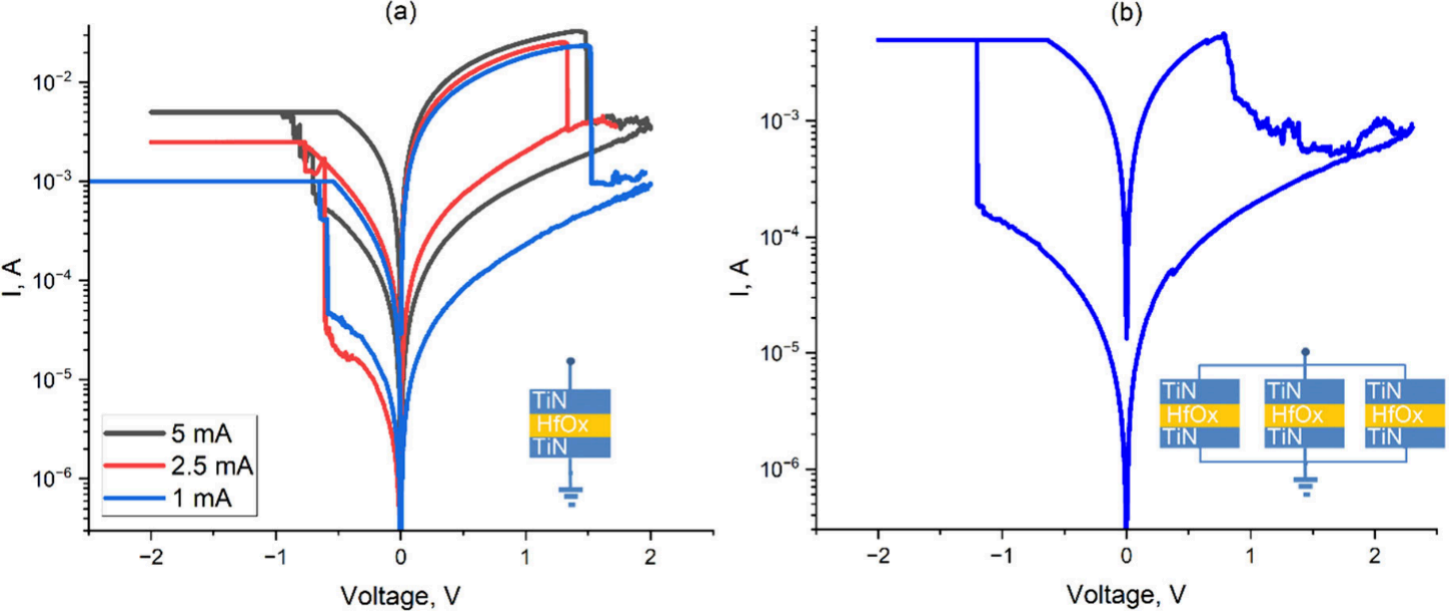
Current–voltage characteristics of individual RRAM cells (a) and after the same cells are connected in parallel (b).

## ΙV Discussion

Intrinsic cycle-to-cycle variability of a metal-oxide-based RRAM cell has been intensively studied through experimental and theoretical research.^13,29,30^ Nonvolatile bipolar resistive switching is believed to rely on the voltage-driven formation/disruption of a single conductive filament. It was shown in refs 13 and 29 that both low- and high-resistance states demonstrate generic statistical variability from cycle to cycle due to the 1D structure of CF and the discrete nature of defects. The model developed in ref 13 predicts the distribution of the Set state as a function of the compliance current during set and the distribution of the Reset state as a function of the stop voltage during Reset. The filamentary mechanism of resistive switching in the RRAM cells based on hafnium and titanium oxides is confirmed by the weak, if any, dependence of cell resistance on its area in a low-resistance state,^31^ thus implying that the area of conductive filaments is notably smaller as compared to the cell area. As the typical thickness of the metal oxide films is a few nanometers, the electric field that initiates drift of oxygen vacancies is rather high (10^6^–10^7^ V/cm). It should be noted that the vertical electric field is not uniform across the cell area due to surface roughness of top and bottom electrodes, variations in the dielectric thickness, and/or concentration of oxygen vacancies.

The typical oxide thickness variation due to roughness of electrodes is 0.5–1 nm.^14^ Thus, such a local nonuniformity of the electric field strength across the cell area is the main reason for the creation of multiple filaments within. Each of the formed filaments contributes to the cell conductivity during both high- and low-resistive states, thus causing cell resistance intrinsic variability from cycle-to-cycle. Another reason for the formation of multiple filaments is delamination of one of the metal electrodes from the active oxide layer as it was demonstrated in ref 32 on Pt/TiO_2_/Pt RRAM cells after switching to a low-resistive state. The formation of multiple conductive filaments was in situ observed in the ZrO_2_ layer using transmission electron microscopy.^22^

In the case of HfO_*x*_ as a material of the functional layer of RRAM, we have shown that the presence of many conductive filaments in the cell worsens the reproducibility of switching parameters and, as a result, limits the number of possible resistive states. Our analytical model developed to explain the observed intrinsic variabilities is based on the assumption that the dominating mechanism of conductivity in hafnium-oxide-based film is direct tunneling. Good agreement between experimental results and the modeling data is obtained under the assumption of the formation of multiple conductive filaments when one filament is dominating by its length and thus providing maximum contribution to the cell conductivity in a low-resistive state, while the contribution of smaller filaments is notable in a high-resistance state. The nonreproducibility of the switching parameters, characteristic of the high-resistance state, arose because the length of the main filament was compared with the length of the secondary filaments in the high-resistance state.

When several formed filaments are rather similar in length, their contribution to conductivity becomes important during transition from a low- to a high-resistance state. Again, multiple filaments can be considered as resistors connected in parallel. During Reset switching the lengths of these filaments will be progressively reduced, thus causing an increase of the local dielectric thickness and decrease of the tunneling current. In the case of the gradual Reset model, there is no main filament, and therefore, there is no influence of smaller filaments that could occur during formation on Reset switching. This key difference between the two models shows why the presence of many filaments in a RRAM cell can be both a disadvantage and an advantage in terms of achieving reliable multilevel states necessary to create analogue neuromorphic systems.

Although experimental study of the HRS resistance variability and the experimental study of a gradual Reset were conducted on different materials (HfO_*x*_ and TiO_*x*_, respectively), the model of multifilamentary switching is applicable for both phenomena in RRAM cells based on different transition metal oxide layers. Filamentary conduction, where the filament itself consists of oxygen vacancies, is reported in many materials, such as HfO_*x*_, TiO_*x*_, ZrO_*x*_, TaO_*x*_, and other transition metal oxides.^10^ The choice of specific materials for experiments described in this paper was based solely on material availability.

## V Summary and Conclusions

In this paper we have addressed two critically important issues related to metal-oxide-based RRAM switching characteristics, namely, intrinsic cycle-to-cycle variability in a high resistance state and sharp vs gradual change of conductivity during transition from a low- to a high-resistance state. In the present work, these issues were studied theoretically and experimentally. It was shown that both issues are related to the formation of multiple conductive filaments, which create parallel conductive paths within a thin dielectric layer. In the case of one dominating by length filament and several smaller filaments, our model predicts rather sharp transition to a low-resistance state and notable variability of resistance from cycle to cycle, thus limiting the number of statistically reliable resistive states. In the case of the formation of several conductive filaments with rather similar size, the change of the conductance during Reset occurs gradually with increasing voltage, thus providing an opportunity to obtain multiple resistive states. Depending on the relative difference of the lengths of the filaments, our model of multifilament switching allows us to explain both the nonreproducibility of resistance in the high-resistance state and the effect of smooth switching in a Reset mode. The effect of several filaments on the reproducibility of resistance in a high-resistance state and on a gradual Reset has also been experimentally verified.

In conclusion, solving the issues of intrinsic cycle-to-cycle variability for a high resistance state and extrinsic cell-to-cell variability of switching during Reset will allow us to build RRAM arrays for neuromorphic applications with improved functionality and reliability.

## Abbreviations

RRAM: Resistive Random-Access Memory
1T1R: 1 Transistor 1 Resistor
MOSFET: Metal-Oxide-Semiconductor Field Effect Transistor
DC: Direct Current
ALD: Atomic Layer Deposition
PVD: Physical Vapor Deposition
LRS: Low-Resistance State
HRS: High-Resistance State
CF: Conducting Filament
COD: Coefficient of Determination
